# Identification of differentially expressed genes in carp rods and cones

**Published:** 2008-02-26

**Authors:** Yoshie Shimauchi-Matsukawa, Yoshinobu Aman, Shuji Tachibanaki, Satoru Kawamura

**Affiliations:** 1 Graduate School of Frontier Biosciences, Osaka University, Osaka, Japan; 2 Department of Biology, Graduate School of Science, Osaka University, Osaka, Japan

## Abstract

**Purpose:**

Rods and cones differ in their photoresponse characteristics, morphology, and susceptibilities to certain diseases. To contribute to the studies at the molecular level of these differences, we tried to identify genes expressed preferentially in rods or cones.

**Methods:**

From purified carp rods and cones, we extracted their RNA and obtained corresponding cDNA pools (rod cDNA and cone cDNA). We employed the suppression subtractive hybridization method to identify the genes expressed preferentially in rods or cones. Cone cDNA was subtracted from rod cDNA to obtain cDNA, which ideally contained cDNA expressed preferentially in rods (R/c cDNA). Similarly, rod cDNA was subtracted from cone cDNA to obtain C/r cDNA. With differential array screening, we screened candidate genes that were expressed mainly or exclusively in rods or cones. The nucleotide sequences of the positive genes were determined. In some of them, their mRNA localizations were confirmed by in situ hybridization.

**Results:**

R/c cDNA contained genes already known to code rod specific proteins, such as cGMP gated channel, transducin β1, and rhodopsin. In sharp contrast, C/r cDNA contained genes that code proteins of which functions are mostly unknown. Among them, N-myc downregulated gene 1-like (NDRG1L) and aryl hydrocarbon receptor 2 (AhR2) were most abundant, and by in situ hybridization, they were proven to be expressed specifically in cones.

**Conclusions:**

Using purified rods and cones, we identified mRNAs expressed preferentially in rods or cones. Of particular interest is the specific expression of NDRG1L and AhR2 in cones.

## Introduction

Rods and cones show different photoresponse characteristics: the light sensitivity is high in rods but low in cones, and the time resolution is low in rods but high in cones [1]. They are also morphologically different: the outer segment of a rod consists of stacks of disc membranes surrounded by its plasma membrane, while that of a cone consists of tightly stacked lamellae of the plasma membrane. Rods and cones are also different in their synaptic structures [2] and retinomotor movement in response to light [3]. In addition to these biologic differences, they show distinct susceptibilities to certain diseases [4]. All of these variances probably arise from the differences in the amount as well as the types of proteins expressed in rods and cones.

In previous studies, several attempts were made to identify rod-enriched genes in mouse [5] and human fovea genes potentially enriched with genes expressed in cones [6]. Recently, using Nrl^−/−^ mice lacking rods but expressing predominantly S-cones [7,8], the gene expression pattern was compared between wild-type (rod) and Nrl^−/−^ (S-cone) mouse retina [9,10]. In our present study, we took a different approach to identify the genes preferentially expressed in rods or cones by using purified rods and cones isolated from wild-type carp retina.

We previously succeeded in purification of rods and cones from carp retina [11,12]. In our preparation, we found >75% of purified rods and 10%–20% of purified cones retained the ellipsoid and the myoid. In previous in situ hybridization studies, it has been shown that significant amounts of photoreceptor mRNAs are detected in the myoid and the ellipsoid [13-15]. From the localization of mRNA identified in these studies, we thought that rod and cone mRNA are retained in our purified rods and cones.

In the present study, we extracted RNA from our purified rods and cones in carp and tried to find out the genes preferentially expressed in rods or cones. In contrast to the previous studies in which the expression level of each gene was measured [5,6,9,10], we used the suppression subtractive hybridization (SSH) method. In this method, rod (cone) genes were subtracted from cone (rod) genes to find genes differentially expressed in cones (rods). Our result showed that there are a group of genes preferentially expressed in either rods or cones.

## Methods

### Preparation of carp photoreceptor RNA

Common carp (*Cyprinus carpio*), 25–30 cm in length, were purchased from a local supplier, and kept in dark or light for at least 3 h. Carp were cared for in accordance with our institutional guidelines. Carp rods and cones were purified as described [11,12]. Rods and cones were brushed off the retina in a Ringer’s solution (119.9 mM NaCl, 2.6 mM KCl, 0.5 mM CaCl_2_, 0.5 mM MgCl_2_, 0.5 mM MgSO_4_, 1 mM NaHCO_3_, 16 mM glucose, 0.5 mM NaH_2_PO_4_, 4 mM HEPES, pH 7.5), and the resultant suspension of rods and cones was filtered through a nylon mesh to eliminate large fragments of retinal tissue. The filtrate containing isolated rods and cones was layered on the top of a stepwise Percoll gradient (30/45/60/70/75/90%; w/vol), and centrifuged for 20 min at 10,000 x g. Rods were sedimented at the 45/60% interface and cones were sedimented at the 75/90% interface. These purified rods and cones were collected and mixed with the same volume of the Ringer’s solution to reduce the density of Percoll and centrifuged firstly at 600 x g for 12 s and then at 3,000 x g for 4 s. Cones were washed additionally with a K-gluconate buffer (K-gluc buffer; 115 mM K-gluconate, 2.5 mM KCl, 2 mM MgCl_2_, 0.2 mM EGTA, 0.1 mM CaCl_2_, 1 mM dithiothreitol, 10 mM HEPES, pH 7.5) by centrifugation (600 x g for 12 sec and then 3,000 x g for 4 sec) [16]. After the cells were sedimented by centrifugation, they were collected, rapidly frozen in liquid N_2_, and stored at -80 °C. Our purified cone preparation and rod preparation contained a small amount of hemocytes [11]. Therefore, we also collected hemocytes from carp blood and stored at -80 °C. Total RNA was isolated using a GenElute Mammalian Total RNA Kit (Sigma-Aldrich, St. Louis, MO) as described in the manufacturer’s instruction. In brief, rods, cones or hemocytes kept at -80 °C were thawed and lyzed by addition of 500 μl of a lysis solution supplemented in the kit with added 2-mercaptoethanol, and mixed thoroughly. The cell lysate was transferred to a filtration column attached to the kit to remove cellular debris and to shear DNA. We thus obtained rod RNA, cone RNA, and hemocyte RNA. Chemicals were obtained either from Sigma-Aldrich or nacalai (Kyoto, Japan) unless otherwise indicated.

### Suppression subtractive hybridization

Extracted total RNA was treated with DNase I (Amplification Grade; Invitrogen, Carlsbad, CA) at room temperature for 15 min. Then cDNA synthesis and pre-amplification of cDNA were conducted using a Smart–PCR cDNA Synthesis Kit (BD Biosciences, Franklin Lakes, NJ) to obtain cDNA from a rod preparation (rod cDNA), a cone preparation (cone cDNA), and hemocytes (hemocyte cDNA). To obtain candidate cDNAs that were possibly expressed preferentially in rods, we subtracted cone cDNA from rod cDNA based on the method of SSH using a BD PCR-Select cDNA Subtraction Kit (BD Biosciences) [17]. This method increases the possibility of identifying mRNAs expressed preferentially in rods or cones. The cDNA thus obtained by subtraction of cone cDNA from rod cDNA is termed R/c cDNA. Similarly, to obtain C/r cDNA, we subtracted rod cDNA from cone cDNA. In both cases, hemocyte cDNA was also subtracted. With the SSH method, ideally, all genes expressed in both rods and cones are subtracted. As a consequence, if there are genes preferentially expressed in rods, those genes are detected in the R/c cDNA. Similarly, the genes expressed preferentially in cones are found in the C/r cDNA. (However, careful identification was necessary. See Results and Discussion.) The SSH method causes equalization of high and low abundance mRNAs, and therefore, this method is useful to detect the genes of which expression levels are low. For the same reason, however, an abundant gene—for example, rhodopsin gene in rods—is not often detected, and the number of such a gene is much reduced comparing with that expected from the actual abundance of that gene in a given cDNA library (see Results and Discussion).

The R/c cDNA and the C/r cDNA were purified using a Wizard DNA Clean-up system (Promega, Madison, WI). Purified products (25 ng) were inserted into vectors using a pGEM T Easy Vector System (Promega), and were introduced into *E. coli* XL-10Gold (Stratagene, La Jolla, CA). Transformed cells were cultured on an LB agar plate supplemented with isopropyl β-D-thiogalactoside, 5-bromo-4-chloro-3-indolyl-β-D-galactoside, and ampicillin.

### Differential array screening

To determine the candidate genes expressed preferentially in cones, for example, we randomly selected a total of 576 colonies from the cells transformed by the C/r cDNA (C/r cDNA transformants). C/r cDNA fragments in individual transformants were amplified by PCR with T7 and SP6 primers. PCR reactions were performed at 94 °C for 1 min, then at 55 °C for 1 min, and finally at 72 °C for 1.5 min with Taq polymerase (Takara, Ohtsu, Japan). This sequence was repeated for 30 cycles. PCR products were treated with ExoSAP-IT (USB, Cleveland, OH) to remove unconsumed dNTPs and primers, and 1 µl of each treated PCR product was manually spotted on two sheets of Hybond-N+ membrane (GE Healthcare, Piscataway, NJ) at identical positions. These membranes were air-dried and exposed to ultraviolet light for DNA fixation. The two membranes were probed by the rod or the cone cDNA to discern the candidate genes expressed preferentially in cones. Probes were prepared from the rod and the cone cDNA synthesized by the Smart–PCR cDNA synthesis kit (see previous section), and they were labeled with digoxigenin (DIG) by using DIG DNA Labeling Mix (Roche Diagnostics, Basel, Switzerland). Two membranes were hybridized with either DIG-labeled rod or cone cDNA. These membranes were washed under high-stringency conditions: twice with 2 x SSC and then twice with 0.5 x SSC, both in the presence of 0.1% SDS at 50 °C. The hybridized DNA probes were detected by anti-DIG-POD Fab fragments (Roche Diagnostics) and visualized with Chemi-Lumi One (nacalai). The hybridization signal in each spot was quantified using a LAS-1000 imaging system (Fuji Film, Tokyo, Japan), and background signals were subtracted. This hybridization screening was repeated twice by using cDNA probes prepared independently.

**Figure 1:**
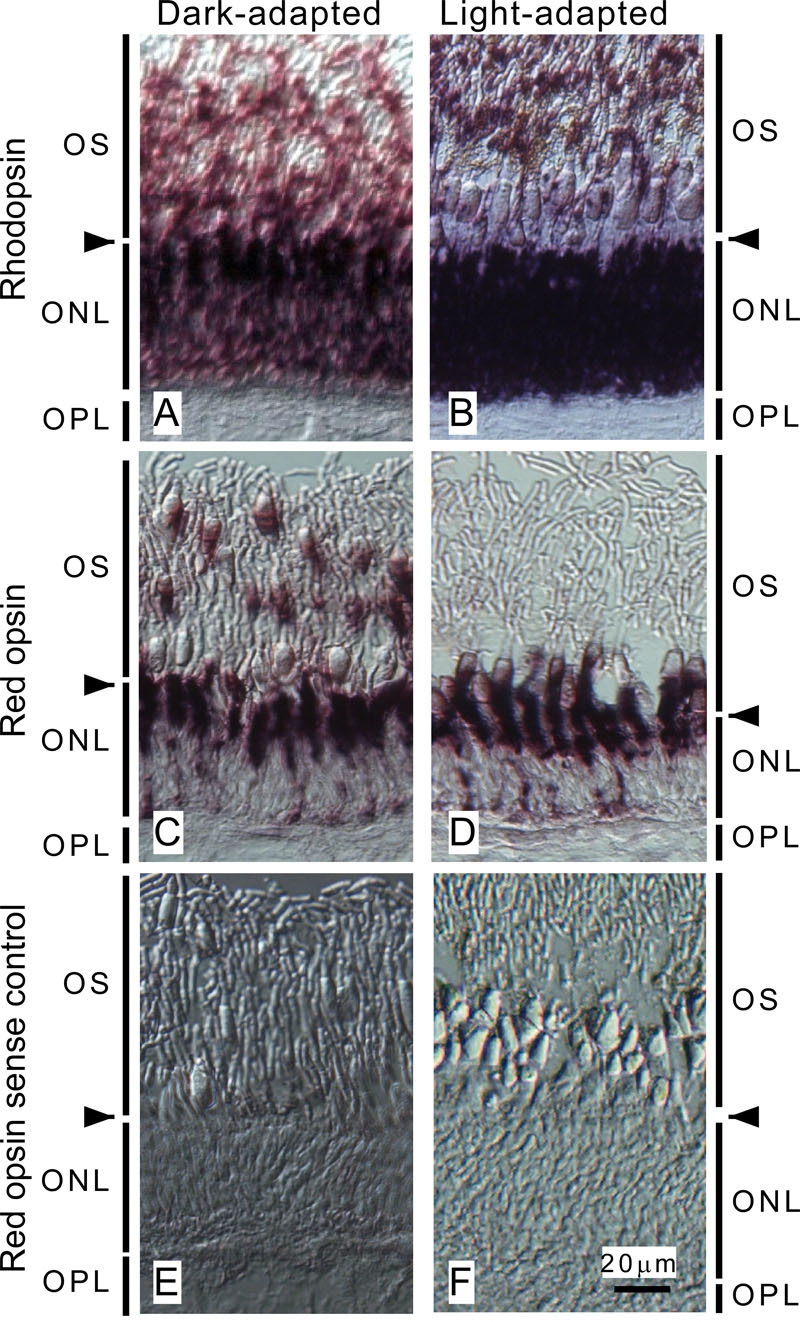
Distribution pattern of opsin mRNA in carp photoreceptor cells. Antisense cRNA probes were hybridized with mRNA of rhodopsin (**A** and **B**) and that of red-sensitive opsin (**C** and **D**) in dark-adapted (**A** and **C**) and light-adapted (**B** and **D**) retina. **E** and **F**: Controls were obtained using sense probes of red opsin in dark-adapted (**E**) and light-adapted (**F**) retina. Positive signals of rhodopsin in the outer segment layer in **A** and **B** are from the ellipsoid and the myoid of rods extending distally. Arrowheads indicate the approximate positions of the outer limiting membrane. The following abbreviations were used: outer segment layer (OS), outer nuclear layer (ONL), and outer plexiform layer (OPL). Bar indicates 20 μm in **F**.

To determine the candidate genes expressed preferentially in rods, we performed a screening similar to that employed for cones but using cDNA fragments obtained from R/c transformants. The PCR product was spotted on a pair of membranes and was probed by the DIG-labeled rod and cone cDNA.

### Sequence analysis

The candidate cDNA identified in the aforedescribed differential array screening was used as the template DNA for the sequence analysis. Nucleotide sequences were determined with an ABI Prism 3100-Avant Genetic Analyzer (Applied Biosystems, Foster City, CA) using BigDye Terminator v3.1. Sequence homology searches were performed using the Blast N program at NCBI.

### In situ hybridization

To confirm that the aforedescribed candidate genes that we identified were actually expressed preferentially in rods or cones, we employed in situ hybridization to examine the expression levels of the corresponding mRNAs in rods or cones. The candidate cDNA fragments were amplified with T7 and SP6 primers by means of colony PCR using the corresponding glycerol stock of *E. coli*. Amplified DNA was purified by phenol-chloroform extraction twice followed by chloroform extraction, and then used as the template for in vitro cRNA transcription. cRNA riboprobes were synthesized by run-off transcription from the SP6 or T7 promoter with DIG RNA Labeling Mix (Roche Diagnostics) according to the manufacturer’s instruction.

**Figure 2:**
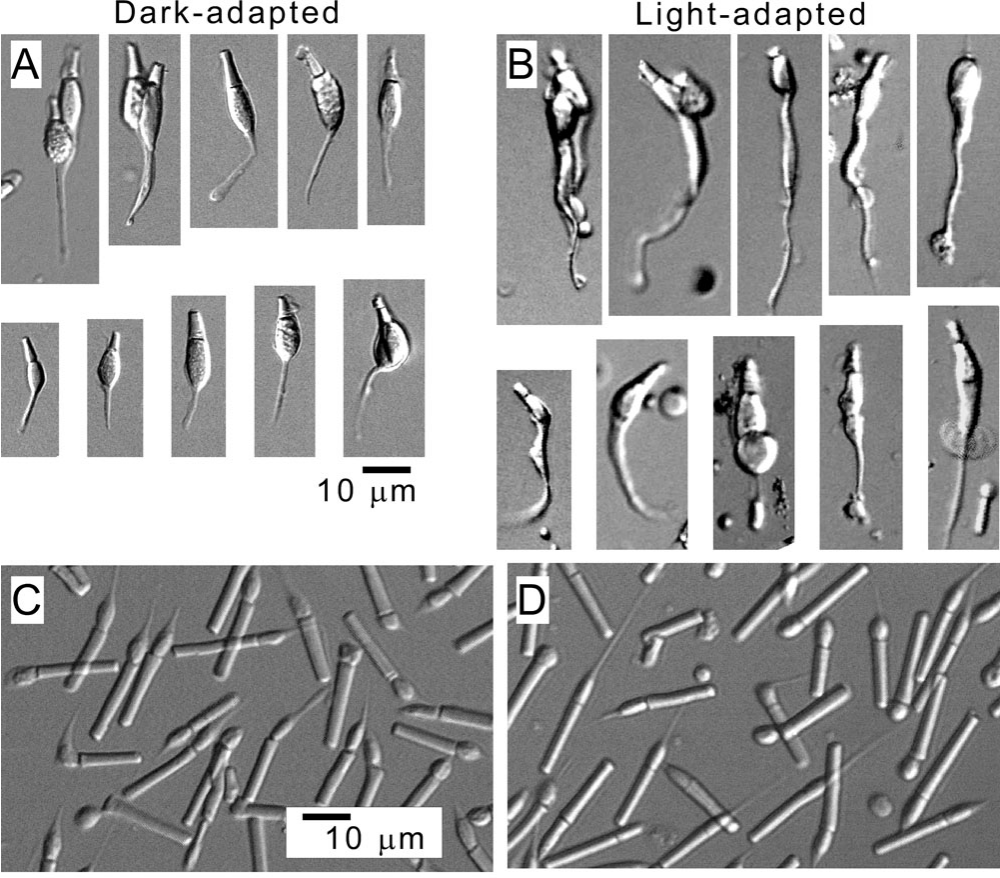
Morphology of photoreceptors isolated from dark- and light-adapted retinas. Cone cells were isolated from dark-adapted (**A**) and light-adapted retina (**B**). In both (**A**) and (**B**), ten typical cells are shown. Rods were isolated from dark-adapted (**C**) and light-adapted retina (**D**). Bars indicate 10 μm.

In situ hybridization was performed as described previously [15]. The sections were dried at 50 °C for 15 min, soaked in chloroform for 5 min, and air-dried at room temperature. The sections were post-fixed with 4% paraformaldehyde in a 0.1 M phosphate buffer (pH 7.4) for 30 min and then treated with 0.2 N HCl for 10 min. Acetylation was carried out in 0.1 M triethanolamine (pH 8.0) supplemented with 0.25% acetic anhydride for 10 min. Proteinase K (10 μg/ml) treatment was carried out for 30 min at room temperature. The sections were hybridized with 0.1-2.0 μg/ml cRNA probes. The hybridization buffer contained 50% formamide, 6 x SSC, 5 x Denhardt's solution, 0.1 mg/ml yeast tRNA, and 1μM EDTA. The hybridization signal was visualized with an alkaline phosphatase reaction. In each observation of the hybridization signal, we performed a control experiment with a sense probe in parallel with the antisense probe under the same conditions. When detectable signals were found in the control experiment, the results of the antisense probes were discarded.

## Results

### Preparation of photoreceptor cells suitable for RNA extraction

We used mechanically dissociated rods and cones from carp retina to compare the phototransduction cascade between rods and cones [11,12]. Our rods and cones retained the ellipsoid densely packed with mitochondria and the myoid containing endoplasmic reticulum and Golgi apparatus, but they did not retain the nuclei. The proximal part of the ellipsoid and the myoid are the major sites where mRNAs were detected [13-15]. As can be seen in Figure 1, rhodopsin mRNA was detected mostly throughout the outer nuclear layer where rod ellipsoid and myoid region were located (Figure 1A and B), and red opsin mRNA was detected mostly in the cone ellipsoid and myoid (Figure 1C and D). For this, we thought that we could isolate mRNA from our purified rods and cones. We were successful in isolating mRNA from our rods [15], but in contrast, we found it difficult to isolate mRNA from our cones. The difficulty of isolation of cone mRNA was thought to be due to low content of mRNA in our cone preparation as well as low yield of the purification of cones. To overcome this problem, we sought to determine the conditions necessary to obtain cone cells that have more mRNA than those we had prepared.

**Figure 3:**
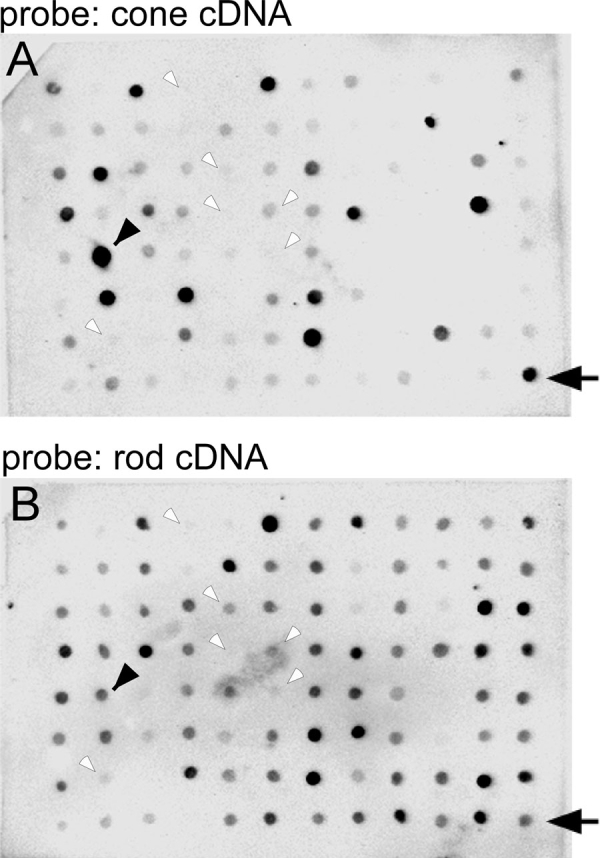
Differential array screening. Clones in the C/r cDNA were spotted on two membranes at the identical positions, and they were hybridized with the cone (**A**) or the rod (**B**) cDNA. A pair of arrows or arrowheads indicates the candidate cDNA clone that is expressed preferentially in cones. As controls, plasmid vectors were spotted (small empty arrowheads).

In fish, rods and cones are known to show retinomotor movements: under dark-adapted conditions, the myoid is contracted in rods while it is elongated in cones, and under light-adapted conditions, it is contracted in cones but elongated in rods [18]. Our in situ hybridization analysis showed that mRNA was retained in the ellipsoid and myoid in rods and cones both in the dark-adapted and light-adapted retina (rods, Figure 1A and B; cones, C and D). As can be seen in Figure 1D, the red-sensitive cone pigment mRNA was condensed more in the contracted myoid in the light-adapted retina than in the elongated myoid in the dark-adapted retina (Figure 1C). We previously used dark-adapted retina to isolate cones mechanically, and rods and cones were presumably dissociated from the retina at the outer limiting membrane (Figure 1, arrowheads). Therefore, it was possible that our previous purified cones retained elongated thin myoid in which the mRNA content was very low. Because light-adapted cones retained thick myoid that probably contained more mRNA, we isolated cones from light-adapted retina in the present study.

In our previous purification of cones from dark-adapted retina, 10%–20% of them retained the myoid. Similar portions of cones retained the myoid when cones were purified from light-adapted retina. However the morphology of the myoid was remarkably different depending on the adaptation condition. The myoid was short and thin when cones were isolated from a dark-adapted retina (Figure 2A), while it was long and thick when the cells were isolated from a light-adapted retina (Figure 2B). Thus, we extracted RNA from cones purified from light-adapted retina. The myoid of our rods was slightly thicker when the dark-adapted retina was used, but the difference was not so significant (Figure 2C and D).

### Subtraction of photoreceptor cDNA and differential array screening

To identify the genes preferentially expressed in rods or cones, we extracted RNA from a cone preparation that contained 2.7 × 10^6^ cone cells contaminated with <14% hemocytes (cone RNA). RNA was extracted also from a rod preparation (rod RNA) containing 1.1 × 10^8^ rod cells contaminated with 4% cones. Note that the number of cells that could be used, and thus the amount of total RNA extracted, was much lower in our cone preparation than our rod preparation: this was due to a difficulty of purification of large quantities of cones [11]. Then, the corresponding cDNAs were synthesized (rod cDNA and cone cDNA), and they were used for SSH (see Methods). Cone cDNA was subtracted from rod cDNA to obtain R/c cDNA that contained the candidate genes expressed preferentially in rods. Similarly, rod cDNA was subtracted from cone cDNA to obtain C/r cDNA that contained the candidate genes expressed preferentially in cones.

To evaluate the efficiency of the cDNA subtraction, we compared the transcript levels of contaminated cDNA of rhodopsin in the cone cDNA and the C/r cDNA. (Our cone preparation was residually (<1%) contaminated with rods [11].) For this, we compared the difference of the number of amplification cycles necessary to yield a similar amount of the PCR product. The level of the transcript of rhodopsin cDNA was lower with the amount equivalent to 4–5 PCR cycles in the C/r cDNA than in the cone cDNA, which indicated that rod contamination was reduced to 1/20–1/30 in the C/r cDNA. Similarly we compared the levels of red-sensitive opsin cDNA (specific in red-sensitive cones) in the cone cDNA and in the C/r cDNA. The level of red opsin cDNA was found to be higher by 2–3 PCR cycles in the C/r cDNA than in the cone cDNA. From these results, we estimated that cone specific cDNAs were condensed approximately by 60-250-fold in the C/r cDNA after the subtraction. Similar condensation of rod-specific cDNAs was observed in the R/c cDNA (data not shown).

As stated in the previous section, our rod and cone preparations were not perfectly pure and, in addition, the available quantity of cone total RNA was minimal. For these reasons, we carefully checked the results of the subtraction to exclude the false positive signals: we conducted two-step verifications to find out the genes expressed preferentially in rods or cones. First, differential array screening was performed. This step was recommended to perform by the manufacturer, because the SSH method usually introduces false positive signals.

After introduction of R/c cDNA and C/r cDNA into *E. coli*, we randomly picked up 576 colonies from each of R/c cDNA transformants and C/r cDNA transformants. The cDNA fragment in a transformant was amplified by a PCR reaction, and the product was spotted at the identical position on a pair of membranes. Pairs of spots were probed by either the rod cDNA or the cone cDNA (Figure 3). If a spot of C/r cDNA on the membrane gave a higher signal when probed with the cone cDNA than with the rod cDNA (see arrows and arrowheads in Figure 3), it meant that the corresponding gene was present in the C/r cDNA even after subtraction. In other words, that gene was not present or its expression level was low in the rod cDNA so that it remained in the C/r cDNA after the subtraction. Our carp rods and cones were not from an inbred carp strain, and it was possible that some of these clones originated from different alleles. To avoid this possibility, the differential array screening was repeated twice using cDNA probes prepared independently from a different group of carp. In the case when both studies gave similar high positive signals, the gene was thought to be the candidate that is expressed preferentially in rods or cones. We obtained 137 candidate cDNA clones that were thought to be expressed preferentially in rods and 46 candidate clones thought to be expressed preferentially in cones. The sequences of these genes were determined, and the searched results are summarized in Table 1. As controls, plasmid vectors were spotted on the membrane and probed (empty arrowheads in Figure 3). Their signal intensities were much lower than those of spots used for the studies described in the next section.

**Table 1:** Summary of the result of Blast homology searches

	Rod clones	Rod contiguous clones	Rod genes identified	Cone clones	Cone contiguous clones	Cone genes identified
Clones sequenced	137			46		
Empty plasmids	2			1		
Multiplicate sequences	23			5		
Query sequences	112			40		
Blast N hit	87	31	27	24	18	16
No hit	25	21		16	12	

Data are indicated in number of clones. In the differential array screening (Figure 3), the positive clones that were found to be expressed preferentially in rods (Rod clones) or cones (Cone clones) were picked up. The nucleotide sequences of these clones were determined (Clones sequenced). Except for the clones of empty plasmids (Empty plasmids) and those found multiplicate (Multiplicate sequences), each clone was searched using the Blast N program (Query sequences). Among the genes found in the databases (Blast N hit), candidate genes specifically expressed in rods (Rod genes identified) and in cones (Cone genes identified) were identified. In the search, some of the clones were not found in the databases (No hit), and contiguous sequences were also found (Rod and Cone contiguous clones).

### Sequence analysis of candidate cDNA clones preferentially expressed in rods

The sequences of 137 candidate cDNA clones were searched against public sequence databases using the Blast N program. The complete list of the genes is shown in Table 2. Among the 137 clones, there were six genes known to be present in rods: cyclic nucleotide gated channel A subunit (44 clones), transducin β subunit (eight clones), rhodopsin (two clones), GRK1A-1a (two clones), GRK1A-1b (one clone), and transducin γ subunit (one clone). There were 11 other candidates, and among them, the gene similar to aspartate β-hydroxylase isoform b was most abundant (five clones). There were another ten clones that were hit with genomic sequences. However, 25 clones were not hit with any sequences in the database (Table 1).

**Table 2:** Identified rod genes

GI	Identification [gene symbol]	Sp	Length (bases)	Lowest Expected value	Start - End (position of a base)	Signal Intensity	Frq	ISH
	**Known as rod specific genes**							
27542822	Cyclic nucleotide gated channel [gfCNG3]	Ca	2654	0.E+00	1658 - 2397	29.4	44	r1
34785175	Guanine nucleotide binding protein (G protein), beta 1 subunit [gnb1]	Dr	3230	1.E-36	2155 - 2255	5.2	8	
765276	Rhodopsin [rhodopsin]	Cc	1584	0.E+00	579 - 1181	5.9	2	
12862624	G protein-coupled receptor kinase 1A-1a [GRK1A-1a]	Cc	3777	1.E-98	2036 - 2289	11.2	2	
83955365	G protein-coupled receptor kinase 1A-1b [GRK1A-1b]	Cc	3565	1.E-98	2667 - 3040	16.9	1	
37748508	Guanine nucleotide binding protein (G protein), gamma 1 subunit [gngt1]	Dr	704	9.E-39	166 - 310	29.6	1	
	**Other genes**							
68356047	Similar to aspartate beta-hydroxylase isoform b [asph]	Dr	3592	1.E-17	3106 - 3179	19.8	5	r2
29436543	zgc:56703/ DnaJ (Hsp40) homolog, subfamily C, member 5 [zgc:56703]	Dr	2556	3.E-18	1684 - 1845	5.7	4	r3
56967377	O-GlcNAc transferase [OGT]	Dr	3111	0.E+00	778 - 1376	3.5	1	r4
89886286	Facilitated glucose transporter 1 [slc2a1]	Dr	1696	4.E-102	1167 - 1521	4.0	1	r5
68356301	Hypothetical protein LOC554424/ Polo-like kinase 3 [LOC554424]	Dr	3289	5.E-74	294 - 513	28.4	1	
42794004	ADP-ribosylation factor 2 [arf2]	Dr	2660	6.E-15	1878 - 1970	6.6	1	
50368934	l-isoaspartyl protein carboxyl methyltransferase [pcmt]	Dr	2740	1.E-17	2625 - 2723	15.8	1	
28278430	Profilin 2 like [pfn21]	Dr	2020	2.E-53	1309 - 1497	3.3	1	
41055366	zgc:56668/ Signal recognition particle 9 kDa protein [srp9]	Dr	757	2.E-78	96 - 422	13.0	1	
68392381	Nuclear factor of kappa light polypeptide gene enhancer in B-cells inhibitor-like 1 [nfbbil1]	Dr	7291	1.E-47	7101 - 7223	1.9	1	
18147599	Carbonic anhydrase 2 [Car2]	Th	1976	1.E-25	1259 - 1452	10.4	1	
	**Hit with genomic sequence**							
62868299	Zebrafish DNA sequence from clone DKEY-31K5 in linkage group 8	Dr	87522	3.E-34	78867- 78956	11.9	2	
40353178	Zebrafish DNA sequence from clone CH211-235E18 in linkage group 2	Dr	196642	2.E-60	177487 - 177740	2.0	1	
54606605	Zebrafish DNA sequence from clone CH211-194G1 in linkage group 5	Dr	197143	5.E-102	100810 - 101064	7.0	1	
54606605	Zebrafish DNA sequence from clone CH211-194G1 in linkage group 5	Dr	197143	1.E-63	99403 - 99645	10.4	1	
73853724	Zebrafish DNA sequence from clone CH211-103A8 in linkage group 3	Dr	165773	4.E-11	91117 - 91175	3.0	1	
40994808	Zebrafish DNA sequence from clone CH211-206I14 in linkage group 7	Dr	173867	9.E-40	112044 - 112153	1.7	1	
50724681	Zebrafish DNA sequence from clone DKEY-100E19 in linkage group 14	Dr	262038	8.E-21	228195 - 228365	4.6	1	
55818874	Zebrafish DNA sequence from clone DKEY-158P11 in linkage group 4	Dr	92140	3.E-12	15398 - 15466	24.5	1	
42517023	Zebrafish DNA sequence from clone DKEY-208P1 in linkage group 17	Dr	188037	4.E-94	92142 - 92399	12.1	1	
51127565	Zebrafish DNA sequence from clone DKEY-95O3 in linkage group 15	Dr	155540	2.E-13	73341 - 73448	4.5	1	

The results of homology search analysis are summarized. Candidate genes preferentially expressed in rods were searched in our differential array screening (similar study as shown in Figure 3), and the sequence of a clone obtained in our study was compared with those of the known genes in the database. The gene that showed the highest homology to our clone is listed with its gene identification number (GI), the name of identified gene (Identification [gene symbol]), animal species of the gene (Sp), and the length of the gene (Length). The region of our clone in the gene identified is shown as the position of a base in the gene (Start-End) together with an index to show the degree of homology (Lowest expected value). Also shown are a signal intensity (a mean of 2 independent studies) of rod cDNA relative to that of cone cDNA (Signal Intensity), the number of clones we identified (Frq), and the label of the in situ hybridization study shown in Table 4 (ISH). Species are: Ca, *Carassius auratus*; Cc, *Cyprinus carpio*; Dr, *Danio rerio*; Th, *Tribolodon hakonesis.*

### Sequence analysis of candidate cDNA clones preferentially expressed in cones

The sequences of 46 candidate cDNA clones were searched similarly and are summarized in Table 3. Interestingly, we did not detect genes that are known to be expressed specifically in cones. For example, we did not find the mRNA of red-sensitive opsin in the C/r cDNA (see Discussion). Instead, we identified N-myc downstream regulated gene 1-like gene (NDRG1L; four clones) and aryl hydrocarbon receptor 2 (AhR2) gene (four clones) as the most abundant genes in the C/r cDNA. We also found ten known genes (one clone each), two housekeeping genes, mitochondrial ATPase 6 (three clones), and eukaryotic translation initiation factor 5 (one clone). There were another two genes that were hit with genomic sequences. We also found that 16 clones were not hit with any sequences in the database (Table 1).

**Table 3:** Identified cone genes

GI	Identification [gene symbol]	Sp	Length (bases)	Lowest Expected value	Start - End (position of a base)	Signal Intensity	Frq	ISH
	**Other genes**							
37589638	N-myc downstream regulated gene 1, like [ndrg1l]	Dr	1712	0.E+00	55 - 733	5.3	4	c1
18858260	Aryl hydrocarbon receptor 2 [ahr2]	Dr	7126	4.E-30	5574 - 5680	2.3	4	c2
46249950	zgc:85611/Acyl-coemzyme A binding domain containing [zgc:85611]	Dr	1745	6.E-48	442 - 630	2.5	1	c3
42542723	14-3-3 theta polypeptide/ ywhaq [ywhaq]	Dr	1943	1.E-87	1055 - 1467	1.5	1	c4
28279778	SET translocation (myeloid leukemia-associated) A [seta]	Dr	1715	0.E+00	315 - 847	3.1	1	c5
62132940	wu:fa20e05/ Spectrin alpha chain, brain [wu:fa20e05]	Dr	1795	0.E+00	761 - 1186	5.1	1	c6
68360189	Similar to Kruppel-like factor 9 [LOC565869]	Dr	987	3.E-73	739 - 985	3.0	1	c7
34784092	Opposite strand transcription unit to Stag3 [gats]	Dr	2295	4.E-27	2005 - 2113	5.7	1	c8
28278872	Cyclin L1 [ccnl1]	Dr	1967	5.E-10	1489- 573	5.3	1	c9
39645455	Prosaposin [psap]	Dr	2390	4.E-73	999 - 1329	2.1	1	
19068029	Growth hormone protein gene [gh]	Cal	11576	2.E-16	3370 - 3453	12.1	1	
39645429	Poly A binding protein, cytoplasmic 1 b [pabpc1b]	Dr	2794	5.E-111	1043 - 1416	3.2	1	
	**Housekeeping genes**							
55071759	ATPase 6 gene [mt-atp6]	Cc	668	8.E-99	87 - 271	3.0	3	
85720010	Eukaryotic translation initiation factor 5 [eif5]	Ip	759	1.E-75	197 - 599	1.7	1	
	**Hit with genomic sequence**							
111153896	Zebrafish DNA sequence from clone DKEYP-10C7 in linkage group 23	Dr	171155	1.E-14	20126 - 20236	3.2	1	
52421057	Zebrafish DNA sequence from clone DKEY-19E4 in linkage group 6	Dr	209866	1.E-20	200719 - 200809	3.0	1	

The results of homology search analysis are summarized. Candidate genes preferentially expressed in cones were searched in our differential array screening (Figure 3), and the sequence of a clone obtained in our study was compared with those of the known genes in the database.The gene that showed the highest homology to our clone is listed with its gene identification number (GI), the name of identified gene (Identification [gene symbol]), animal species of the gene (Sp), and the length of the gene (Length). The region of our clone in the gene identified is shown as the position of a base in the gene (Start-End) together with an index to show the degree of homology (Lowest expected value). Also shown are a signal intensity (a mean of 2 independent studies) of cone cDNA relative to that of rod cDNA (Signal Intensity), the number of clones we identified (Frq), and the label of the in situ hybridization study shown in Table 4 (ISH). Species are: Cal, *Catla catla*; Cc, *Cyprinus carpio*; Dr, *Danio rerio*; Ip, *Ictalurus punctatus*.

### In situ hybridization of candidate genes

**Figure 4:**
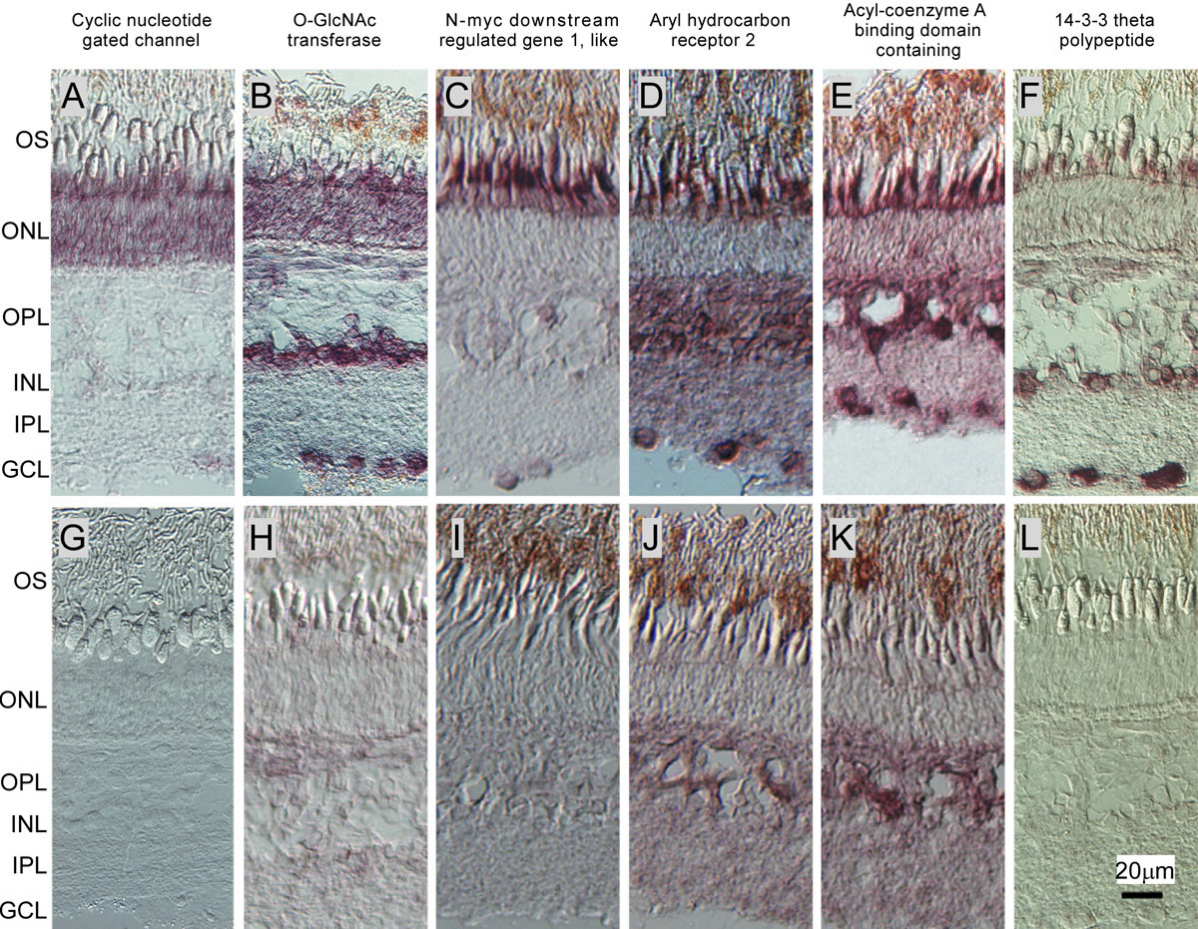
Cellular localization of mRNA of the candidate genes in light-adapted carp retina. Cellular localization of some of the candidate genes was determined with the in situ hybridization (ISH) method. **A** and **B:** ISH signals of rod candidate genes. **A:** Cyclic nucleotide gated channel (r1). **B:** O-GlcNAc transferase (r4). **C-F:** ISH signals of cone candidate genes. **C**: N-myc downstream regulated gene 1-like (c1). **D**: Aryl hydrocarbon receptor 2 (c2). **E:** Acyl CoA binding domain containing (c3). **F:** 14–3–3 Theta polypeptide (c4). **G-L:** Negative controls of A-F with use of sense probes. The following abbreviations were used: outer segment layer (OS), outer nuclear layer (ONL), outer plexiform layer (OPL), inner nuclear layer (INL), inner plexiform layer (IPL), and ganglion cell layer (GCL).

To identify finally the genes that are preferentially expressed in rods or cones, we performed in situ hybridization study using some of the genes in Table 2 (r1-r5 shown in the in situ hybridization column) and in Table 3 (c1-c9). The criterion to identify the cell-type in which a gene of interest is expressed is to determine whether the signal is present throughout the outer nuclear layer (rods) or in the proximal part of cone ellipsoid and in the myoid (cones) as evident in Figure 1. The relative strength of the ISH signals is summarized in Table 4, and some of the in situ hybridization images (r1, r4, and c1-c4) are shown in Figure 4.

**Table 4:** Summary of the result of in situ hybridization analysis of candidate genes

	Rod gene candidates						Cone gene candidates								
	r1	r2	r3	r4	r5		c1	c2	c3	c4	c5	c6	c7	c8	c9
Cone	-	+	-	+	++		+++	++	+++	+	+++	-	-	-	-
Rod	++	-	-	++	-		-	-	+	-	+++	-	-	-	-
Other	-	-	-	++	++		-	+	+++	++	+++	++	+	+	++

We estimated relative intensities of the in situ hybridization signal of the clones found preferentially in rods (r1-r5) and cones (c1-c9). From the results as shown in Figure 4, the intensity was estimated by eye in cones (Cone), rods (Rod) and other cells in the retina (Other), and the signal intensity was classified into 4 groups: very strong (+++), strong (++), moderate (+), and none (-)

The signal of cyclic GMP gated channel A subunit (r1) was exclusively found in the outer nuclear layer (Table 4 and Figure 4A) where rhodopsin mRNA is found (Figure 1A and B). The results, therefore, indicated that this channel is expressed in rods. The expression of O-glucNAc transferase mRNA (r4) was greater in the outer nuclear layer (Table 4 and Figure 4B) than in the cone myoid where the mRNA of red-sensitive opsin is found (Figure 1C and D). The results therefore indicated that this gene is expressed more in rods than in cones. This gene may also be expressed in amacrine cells and ganglion cells (Table 4 and Figure 4B). We observed slight signal of the gene similar to aspartate β-hydroxylase isoform b (r2) unexpectedly in cones but not in rods. Positive signals of DnaJ (Hsp40) homolog, subfamily C, member 5 (r3) were not seen in either rods or cones. Although facilitated glucose transporter 1 (r5) was found in the R/c cDNA, in situ hybridization revealed that it is present in cones and in other retinal neurons (Table 4). These unexpected results were possibly due to low quantity of cone RNA currently available (see Discussion).

In c1-c9, the positive signals were observed exclusively in cones in the case of NDRG1L (c1) (Table 4 and Figure 4C). Therefore, this gene is specifically expressed in cones. Two genes, AhR2 (c2) (Table 4 and Figure 4D) and acyl-CoA binding domain containing (c3) (Table 4 and Figure 4E) showed strong signals in cones and also in other retinal neurons but the signals in rods were weak. One gene, 14–3–3 theta polypeptide (c4), showed weak signals in cones and higher signals in retinal neurons other than cones or rods (Figure 4F and Table 4). The other genes showed strong signals in both rods and cones in addition to other retinal neurons (c5) or retinal neurons other than photoreceptors (c6-c9).

To confirm the preferential expression of NDRG1L and AhR2 in cones, we performed a semiquantitative RT–PCR study using extracted rod and cone RNA. Assuming that we extracted most of RNA from rods retaining myoid (75% of purified rods) and cones possessing contracted myoid (10%–20% of purified cones) and that the amplification efficiency of each PCR cycle was 2, we found that the expression levels of NDRG1L and AhR2 were higher in a cone cell than in a rod cell by roughly 4000 and 500 times, respectively (data not shown). This result confirmed that NDRG1L and AhR2 are preferentially expressed in cones.

## Discussion

In the present study, we tried to identify the genes expressed preferentially in rods or cones. For this, we used the SSH method, and in the R/c cDNA, we obtained several genes already known to be expressed in rods. In the C/r cDNA, we identified one gene (NDRG1L) that is expressed almost exclusively in cones (Figure 4C and Table 4). Three genes (AhR2, acyl-coenzyme A binding domain containing, and 14–3–3 theta polypeptide) were found to be expressed preferentially in cones than in rods, although these genes are also expressed in other retinal neurons. In addition, we found several genes that are possibly expressed preferentially in either rods or cones (Tables 2 and 3).

### Genes expressed preferentially in cones

NDRG1L belongs to the NDR gene family that contains an α/β hydrolase fold but lacks the residues necessary for the hydrolase activity. In addition to NDRG1L, zebrafish has an NDRG1 gene. It is probable that NDRG1L and NDRG1 are derived from duplication of ancestral fish genome based on our molecular phylogenetic tree analysis (data not shown). Zebrafish NDRG1L has been exclusively expressed in retina [19], while NDRG1 is ubiquitously expressed in tissues such as liver, mucous cells, pronephric duct, and retina [20]. In our present study, we showed that carp NDRG1L is specifically expressed in cones among retinal neurons.

NDRG1 was identified as a gene responsible for hereditary motor and sensory neuropathy-Lom [21], which is an early-onset peripheral neuropathy that progresses to severe disability in adulthood. Okuda et al. [22] showed that NDRG1 localizes in the cytoplasm of Schwann cells, and is essential for maintenance of myelin sheaths in peripheral nerves. It has been also reported that this gene is involved in many cellular activities such as stress or hormone responses, carcinogenesis, cell growth and differentiation [23]. NDRG1, a phosphorylated protein, has been shown to move between the cytoplasm and the nucleus. It is possible that NDRG1L is also involved in cone-specific signal transduction.

Aryl hydrocarbon receptor protein (AhR) is known as the dioxin receptor and a ligand-activated transcription factor. AhR has been reported to be duplicated (AhR1 and AhR2) in fish [24]. Our finding that AhR2 is predominantly expressed in cones may indicate that this form of AhR2 has a specific function in cones or that it is expressed in a cell-type specific manner. In zebrafish, AhR2 was knocked-down with morpholino antisense oligos [25]. Injection of this morpholino appeared not to affect normal development but reduced the expression of mRNA of cytochrome P450 1A (CYP1A), the most well characterized target in the AhR signaling pathway. CYP1A is expressed in eye [26] and is postulated to exert its biologic effects in many ways including metabolism of arachidonic acid and production of reactive oxygen [27]. AhR2 may contribute to these reactions through its activation by an intrinsic ligand that remains to be determined. AhR is also known to interact with aryl hydrocarbon receptor-interacting protein (AIP). AIPL1, a possible AIP subtype showing 49% identity to AIP and is associated with Leber congenital amaurosis, is essential for biosynthesis of retinal rod cGMP phosphodiesterase [28]. AhR2 may also have a specific function in cones or it may be expressed in a cell-type specific manner.

In previous studies, the expression level of NDRG1 was compared between wild-type and Nrl^−/−^ mouse retina [9,10]. The expression level of NDRG1 was downregulated in Nrl^−/−^ retina in which rods were substituted by S-cones [7,8]. The expression level of AhR in mouse was similar between wild-type (rod) and Nrl^−/−^ (S-cone) retina [10]. These results are somewhat different from those of our present study: both NDRG1L and AhR2 in carp are expressed preferentially in cones. The difference could be due to the difference in the subtypes (NDRG1L in carp and NDRG1 in mice, and AhR2 in carp and AhR in mice) that may be expressed differently between carp and mice. Similarly, except rhodopsin and transducin β subunit that are known to be rod-specific, most of the genes listed in Tables 2 and 3 are not differentially expressed in rods or S-cones in mice [10]. Because photoreceptor proteins do not always show clear-cut rod/cone expression patterns [29], our results possibly indicate that the comparison of rod/cone expression patterns among different animal species is sometimes confusing.

### Genes expressed preferentially in rods

In our R/c DNA, 44 clones out of 137 clones contained the nucleotide sequence of cyclic nucleotide gated channel A subunit (CNGA). This gene was found in the R/c cDNA derived from our rod preparation (Figure 2C), and in addition, our in situ hybridization study showed that its mRNA was found throughout the outer nuclear layer (Figure 4A) where rhodopsin mRNA is present (Figure 1B). These results strongly suggest that this CNGA is expressed in rods. The CNGA clones obtained in the present study covered 39% - 45% of the entire amino acid sequences of striped bass, mouse, and chicken rod CNGA (CNGA1) and cone CNGA (CNGA3). The amino acid sequences deduced from our clones showed higher identity to mouse and chicken rod CNGA1 channels (71% in mouse and 73% in chicken) than their cone CNGA3 channels (65% in mouse and 68% in chicken), and therefore our carp CNGA is probably a member of the CNGA1 family proteins. However, in striped bass, our clones showed higher identity to a CNGA channel reported to be expressed in cones [30] than that in rods (71% and 63%, respectively). These two striped bass CNGA channels are members of the mammalian CNGA1 family [30]. It will be interesting to determine the CNGA subtype expressed in carp cones.

### Possible origin of false positive signals in suppression subtractive hybridization

Ideally, the R/c cDNA should contain cDNAs expressed preferentially in rods, and the C/r cDNA should contain cDNAs expressed preferentially in cones. Unfortunately not all the candidate genes were found in accordance with this idea (Table 4 and Figure 4). This inconsistency could be caused by any number of technical reasons, but one would be the difficulty in obtaining cone RNA in a quantity large enough to conduct SSH. It is possible that during preparation of cone cDNA used as the probe in our differential screening (Figure 3), some of the genes were not amplified effectively because of their low abundance in the extracted cone RNA. Yet, genes in the rod cDNA could have been amplified in proportion to the level of each gene in the extracted rod RNA. If this is the case, at the stage of the identification of candidate genes with our differential array screening, the signal intensity of a clone that was present more abundantly in the C/r cDNA would be higher when the clone was probed with the rod cDNA than with the cone cDNA. This consideration explains why many of the clones in the C/r cDNA showed higher signals when probed with the rod cDNA than with the cone cDNA (Figure 3). It may also explain why we found many false positive signals in the R/c cDNA (Table 4). Although a gene is expressed preferentially in cones (for example, r5 in Table 4), the amplification of this gene was less efficient in the cone cDNA so that the signal was higher when this clone was probed with the rod cDNA.

In this study, we could not find the genes such as cone red opsin genes known to be specifically expressed in cones. It is probably because the SSH method used in this study equalizes the number of each of the subtracted product (see Methods): in our expressed sequence tag analysis of the mRNA expressed in our purified rods, more than 10% of the rod cDNA (36 clones in 330 clones examined) was the clone of rhodopsin (Aman et al., unpublished results), while in the present SSH analysis, we found only two rhodopsin clones in 112 clones examined (Table 1 and Table 2). It is, therefore, highly probable that we simply did not pick up the red opsin gene in our present analysis. Nonetheless, after careful examinations, we could identify some of the genes almost exclusively (c1 and c2) and preferentially (c3 and c4) expressed in cones.

In summary, we found several genes that are preferentially expressed in rods or cones with the SSH method under the limited condition of low availability of cone RNA. Although we did not study the localization of all of the mRNAs in our list (Table 2 and Table 3), it is highly possible that genes in these lists other than r1-r5 and c1-c9 are expressed exclusively or preferentially in rods or cones. Because the functional role of NDRG1L or AhR2 is not known in cones, the studies on these proteins will be of particular interest.
